# Foreign language learning can improve response inhibition in individuals with lower baseline cognition: Results from a randomized controlled superiority trial

**DOI:** 10.3389/fnagi.2023.1123185

**Published:** 2023-03-23

**Authors:** Judith Alina Grossmann, Steffen Aschenbrenner, Birgit Teichmann, Patric Meyer

**Affiliations:** ^1^Network Aging Research, Heidelberg University, Heidelberg, Germany; ^2^Department of Clinical Psychology and Neuropsychology, SRH Clinic Karlsbad-Langensteinbach, Karlsbad, Germany; ^3^School of Applied Psychology, SRH University Heidelberg, Heidelberg, Germany; ^4^Department for General and Applied Linguistics, Heidelberg University, Heidelberg, Germany

**Keywords:** executive attention, executive functions, cognitive reserve, bilingualism, cognitive training

## Abstract

**Introduction:**

The world’s population is aging, increasing the prevalence of dementia. Recently, foreign language learning in later life has been suggested to improve cognition and thus support healthy cognitive aging. To date, however, there are only a few studies with conflicting findings. Therefore, the purpose of this study was to examine whether learning a foreign language can improve executive attention and executive functions in healthy older adults. Additionally, we sought to identify factors affecting cognitive change in foreign language learners, such as cognitive reserve, previous foreign knowledge and usage, and global cognition at baseline.

**Methods:**

In a randomized-controlled trial, we assigned 34 monolinguals between the ages of 65 and 80 to a language learning or a waiting list control group. The participants enrolled in a Spanish course for beginners that met five days a week for 1.5 h for a total of 3 weeks. The waiting list control group received no intervention but had the opportunity to join the language training at the end of the study. All participants underwent an assessment of executive attention (primary outcome), executive functions, verbal fluency, and attention (secondary outcomes) before, immediately after the course, or after a waiting period of 3 weeks for the control group and 3 months after the course or the waiting period.

**Results:**

Foreign language learning did not significantly improve primary or secondary outcomes, neither immediately nor 3 months after the course. However, moderation analyses revealed that participants with lower global baseline cognition tended to improve more on response inhibition than individuals with higher baseline cognition. This relationship was not evident in the waiting list control group.

**Discussion:**

Our results suggest that studying a foreign language does not generally improve executive attention or executive functioning. Nevertheless, individuals with poorer baseline cognition may benefit cognitively from foreign language learning in response inhibition, a domain particularly affected by cognitive aging. Our findings highlight the need of focusing dementia prevention efforts on groups that are more vulnerable to cognitive decline. Additionally, more individualized approaches, including utilizing technology-assisted learning, might enable participants to practice at their performance level, increasing the likelihood of discernible cognitive gains.

**Clinical trial registration:**

https://drks.de/search/en, identifier DRKS00016552.

## Introduction

Dementia is among the most prevalent causes of disability and dependency in older age and is the seventh leading cause of death. Currently, over 57 million people worldwide are living with dementia. This number is predicted to nearly triple by 2050, turning dementia prevention into one of the most significant health challenges of our time (Schwarzinger and Dufouil, 2022). One of the most promising non-pharmacological approaches to dementia prevention is postulated by the concept of cognitive reserve (CR). The CR hypothesis states that lifetime experiences or activities associated with improved cognitive performance contribute to CR and consequently reduce dementia risk (Stern, 2013). Educational attainment is one of the most significant experiences, and equally, occupational status and engagement in cognitively stimulating activities are usually regarded as markers of CR (Opdebeeck et al., 2016).

During the past two decades, a substantial body of research has also focused on the beneficial effects of bilingualism on cognitive functions in older age (Lehtonen et al., 2018). Although controversial, recent reviews suggest a delay in the onset of dementia symptoms in bilinguals compared to monolinguals of about 4.7 years (Anderson et al., 2020; Brini et al., 2020). Bilingualism – defined as the ability to speak two languages fluently (Bak, 2016) – has also been associated with better cognitive performance, particularly in the domain of executive functions (EF) (Lehtonen et al., 2018; Donnelly et al., 2019; Monnier et al., 2021; Degirmenci et al., 2022). EF is an umbrella term that combines multiple higher-order cognitive processes (Goldstein et al., 2014) involved whenever goal-directed thoughts or control of feelings or behavior are required, e.g., when solving a problem. The so-called bilingual advantage in EF is assumed to result from the constant need for language control during bilingual language processing as both languages are always activated in the brain regardless of the one used (Kroll et al., 2015). Therefore, language control is necessary during flexibly switching between languages or effectively suppressing interference from one language when speaking in the other. This line of research, together with the observation that cognitive training can enhance cognitive functions in older adults (Nguyen et al., 2019), gave rise to the idea of proposing foreign language training in older age as an intervention to stimulate cognitive activity (Antoniou et al., 2013). New language acquisition, while challenging, is still possible for older adults (Kliesch et al., 2018) and could subsequently offer a promising way to augment CR and thus contribute to healthy cognitive aging.

Despite apparent differences between foreign language learning and bilingualism, e.g., in terms of language proficiency, foreign language learning might entail cognitive benefits that overlap with those of bilingualism. However, which cognitive domains foreign language learning is addressing remains debated. More recent theories suggest that bilingual language control is organized hierarchically (Branzi et al., 2016). Correspondingly, language control may be fundamentally rooted in the attentional domain, more specifically in executive attention (Bialystok, 2017), which can be seen as the underlying performance on a broader range of EF tasks (Hilchey and Klein, 2011). According to Posner’s and Peterson’s influential model of attention, executive attention is an aspect of attentional control that is particularly necessary in high-conflict situations, e.g., when two languages are competing for processing (Posner and Petersen, 1990). The executive attention network comprises two sub-networks: a cingulo-opercular system, responsible for monitoring behavior, and a frontoparietal/dorsolateral prefrontal system, enabling switching between tasks or mental sets within a task (Petersen and Posner, 2012). Thus, executive attention is regulated by a network involving the anterior cingulate cortex (ACC) and lateral prefrontal areas (Fan et al., 2002), which are also of great importance in the early stage of foreign language acquisition (Pliatsikas, 2020). Therefore, executive attention might be one of the domains most directly addressed by foreign language learning.

However, research on training-related changes in cognition after foreign language acquisition in older adults is still limited. So far, only eleven studies have addressed this subject. Six of these studies reported significant cognitive improvements after learning a foreign language (Bak et al., 2016; Pfenninger and Polz, 2018; Bubbico et al., 2019; Wong et al., 2019; Long et al., 2020; Meltzer et al., 2021). Most notably, these improvements occurred in tasks measuring aspects of executive attention (e.g., the STROOP task, Simon task, Test of Everyday Attention) (Melrose et al., 2017). However, two of the four studies applying these tasks had only a quasi-experimental design (Bak et al., 2016; Long et al., 2020), and one was a pilot study without a control group (Pfenninger and Polz, 2018). Of the five randomized-controlled trials, two found an improvement in global cognition after foreign language learning (Bubbico et al., 2019; Wong et al., 2019). However, in Bubbico et al. (2019), this supposed effect of foreign language learning was due to a decline in performance in the control group. In Wong et al. (2019), the intervention group improved their performance in working memory besides global cognition, but not compared to an active or passive control group.

Yet, it should be noted that all studies varied considerably in methodology, with most studies targeting different and partly non-specific cognitive domains (e.g., global cognition) and interventions ranging in duration and intensity. Other caveats of previous studies are that most had no follow-up period (Ramos et al., 2017; Ware et al., 2017; Pfenninger and Polz, 2018; Bubbico et al., 2019; Valis et al., 2019; Berggren et al., 2020; Long et al., 2020; Meltzer et al., 2021) and did not exclude participants with suspected cognitive impairment (Bak et al., 2016; Ramos et al., 2017; Ware et al., 2017; Pfenninger and Polz, 2018; Long et al., 2020; Meltzer et al., 2021). For a more detailed summary and discussion of the current body of research, see the literature reviews of Pot et al. (2019) and Ware et al. (2021).

To conclude, by now, there is, at best, weak evidence that foreign language learning may improve cognitive functioning in healthy older adults. Therefore, we developed a randomized-controlled trial to determine cognitive characteristics altered by learning a foreign language. We defined measures of executive attention as the primary outcome. As there is still no consensus on cognitive functions involved in foreign language acquisition, we also included a broader range of EF tasks from the domains of updating, flexibility, and inhibition as secondary outcomes. We hypothesized that foreign language learners would show improved cognitive performance in executive attention and executive functions compared to a passive control group immediately (hypothesis 1) and 3 months (hypothesis 2) after taking a foreign language class. As this study is exploratory, we were also interested in factors influencing cognitive progress among foreign language learners. We hypothesized that CR could predict cognitive change (hypothesis 3). As higher levels of CR are associated with better cognitive performance, the intervention might more likely introduce a significant improvement in cognition in those with lower levels of CR. The same might apply to previous foreign language proficiency and usage. Even though our study included only monolinguals, almost every older adult in Germany learned at least one language in school or later in life through courses. Therefore, we considered it important to analyze the possible role of prior foreign language knowledge skills and usage. We assumed that individuals who are less familiar with foreign languages might benefit more from foreign language learning because their brains are less adapted to cognitive control mechanisms of foreign language acquisition and usage than those of individuals with more previous foreign language experience (hypothesis 4). In deviation from the original study protocol published (Grossmann et al., 2021), we added the baseline level of general cognition as a predictor, following more recent findings by Kliesch et al. (2021) (hypothesis 5). In their study, the authors found that language learners with lower baseline cognition improved more on cognitive tasks than those in an active or passive control condition during the first 20 weeks of the intervention. Their finding is in line with the CR hypothesis. As individuals with lower cognitive performance are at higher risk for dementia (Valenzuela and Sachdev, 2006), these individuals may also have more room for improvement when engaging in cognitively stimulating activities. We therefore expected that the level of global baseline cognition would predict cognitive change in executive attention and executive functions in foreign language learners.

## Materials and methods

The protocol for this study has been published (Grossmann et al., 2021). In the following sections, we summarize key aspects of the study protocol.

### Design and setting

The present study was a randomized controlled superiority trial with two parallel groups to investigate the effects of a 3 week foreign language course on executive attention and EF in healthy community-dwelling older adults. We randomly assigned participants in a 1:1 ratio to one of the two study arms: a language learning group (LLG) and a waiting list control group (WLCG). Data were collected at the Network Aging Research (NAR), Heidelberg University, Germany, and the SRH University Heidelberg, Germany. The study protocol followed the Consolidated Standard of Reporting Trials (CONSORT) statement (Moher et al., 2010).

Due to the COVID-19 pandemic outbreak, the trial had to be temporarily suspended in March 2020. Initially, we aimed to resume recruitment as soon as possible. However, given the ongoing COVID-19 pandemic and the “at-risk” population in terms of age enrolled in our study, we decided to end our trial early in July 2020. We believe that the study participants’ overall well-being and best interest should be prioritized.

### Participants

Participants were recruited *via* advertisement (e.g., in local newspapers) and flyers between March 2019 and March 2020. Interested participants could contact the study team and were then interviewed for eligibility *via* telephone screening. We invited participants to a face-to-face screening in case of a positive evaluation. For enrollment in the study, participants had to be aged between 65 and 80 years and speak German as their native language. Exclusion criteria comprised command of Spanish too high to participate in a Spanish class for beginners (A1.1), bilingualism or multilingualism, greater than basic proficiency of any Romanic language, and the presence of cognitive impairment [Cognitive Functions Dementia (CFD); Jahn and Heßler, 2017; *z* ≤ −1.5 in any subtest] or any neurological or psychiatric condition. For all participants who indicated previous contact with the Spanish language, eligibility for the Spanish course was ensured by first asking a list of questions derived from units 1 to 5 of the course book (Goerrissen, 2016). Second, these participants also had to take a placement test during the face-to-face screening (Ernst Klett Verlag, 2004). A comprehensive list of all eligibility criteria can be obtained from the study protocol (Grossmann et al., 2021). For logistical reasons, the study team collected data in three waves. In each wave, an equal proportion of subjects were enrolled in both study arms.

### Randomization and blinding

A researcher not involved in the study conduct generated the randomization sequence using a web-based based randomization system^1^. She formed permuted blocks of random sizes, two, four, six, and eight, with a list length of 20, respectively, to ensure an even distribution of participants in each study arm. Trial implementers were unaware of the individual lists’ randomization sequence and block sizes. Concealment was guaranteed *via* consecutively numbered, sealed, opaque envelopes containing group allocation information for each participant. There was no blinding of participants or staff to the allotted interventions.

### Interventions

This study comprised two trial arms: an LLG and a WLCG.

#### Language learning group (LLG)

In a Spanish course for beginners, the LLG received 1.5 h of group-based language lessons on five weekdays within a period of 3 weeks, resulting in a total of 7.5 h of formal tuition per week. This type of training length and frequency was chosen because the literature suggests that more frequently delivered, albeit shorter interventions may be more cognitively beneficial than longer courses with lower frequency (Bak et al., 2016; Long et al., 2020; Meltzer et al., 2021). The group size was limited to a maximum of ten participants to minimize individual differences in learning pace and to enable the teacher to address all participants appropriately. Lessons were held in Spanish by a specialized teacher. Where necessary, German was employed to explain new vocabulary or grammar. There was no focus on any specific learning method. However, the content of the lessons followed units 1 to 3 of a workbook widely used in adult education (Goerrissen, 2016). The lessons included vocabulary learning, comprehension of written and spoken language, as well as individual and group-based speaking and writing exercises. Apart from attending regular classes, participants completed homework assignments and practiced at home to consolidate newly learned material. More details on the intervention can be retrieved from the study protocol (Grossmann et al., 2021).

#### Waiting list control group (WLCG)

The WLCG initially received no intervention. However, after study completion, the WLCG participated in a control group program consisting of the same 3 week language course as the LLG and a subsequent additional voluntary examination. The program was introduced to lower barriers to participation due to group preferences and to increase the amount of meaningful data.

### Outcomes

Baseline variables, as well as primary and secondary outcomes, are summarized below. The subdivision into primary and secondary outcomes follows the recommendations of the CONSORT statement (Moher et al., 2010). The primary outcomes are of greatest significance, whereas the secondary outcomes are further outcomes of interest. Detailed descriptions of the questionnaires and tasks applied in this trial can be obtained from the study protocol (Grossmann et al., 2021).

#### Baseline variables

Data measured at baseline included socio-demographic variables, medical information related to the eligibility criteria, foreign language knowledge and usage [Language and Social Background Questionnaire (LSBQ); Anderson et al., 2018], and global cognitive functioning using the CFD [test form: S1 (touchscreen operation); Jahn and Heßler, 2017]. The CFD is a comprehensive computer-based test battery comprising eleven tests from five cognitive domains: attention, verbal long-term memory, EF, expressive speech, and perceptual-motor functions. Besides individual test scores adjusted for age and, where possible, sex, and education, an index score indicates overall cognitive functioning. We applied the Cognitive Reserve Index questionnaire (CRIq; Nucci et al., 2012) to assess CR. The CRIq is a semi-structured interview that measures age-adjusted global CR based on three commonly accepted domains of CR: education, occupational activity, and leisure time activity.

#### Primary outcomes

The primary outcome was *executive attention*, which we assessed by two measures: the Stroop Interference Test (STROOP; Schuhfried, 1999) and the Divided Attention, a subtest of the Perception and Attention Function Battery (WAFG; Sturm, 2006b).

The STROOP is a valid, reliable, and widely used measure of selective attention, representing the monitoring aspect of executive attention (Melrose et al., 2017). Our outcome of interest was the “naming interference tendency”, which is obtained by subtracting the median reaction time of the naming baseline condition from the naming interference condition. In the naming interference condition, color words printed in a different color are consecutively displayed on a computer screen (e.g., the word “green” printed in red). The participant must press the color the word is printed in on a response panel and ignore the meaning. Since the dominant response would be to read the word, the task creates a conflict, the so-called interference, between the dominant and the required response. This continuous conflict processing requires executive attention because the natural reaction has to be inhibited (Petersen and Posner, 2012). By subtracting the performance in the baseline condition from the interference condition, the naming interference is adjusted from color-naming processing speed.

The WAFG is a dual-task procedure assessing divided attention and represents the switching aspect of executive attention (van Zomeren and Brouwer, 1994; Petersen and Posner, 2012). The outcome was the logarithmic mean reaction time to either two consecutive squares or two consecutive high-pitched sounds presented on a computer screen. In this task, reaction time costs arise from coordinating the parallel processing of stimuli from two separate channels. In the visual channel, either triangles or squares appear, and low- or high-pitched sounds emerge in the auditory channel. At the same time, attention must remain focused on the two targets (two squares or two high-pitched sounds).

#### Secondary outcomes

As secondary outcomes, performance on a broad range of tasks from the three core domains of EF - inhibition, shifting, and updating – was assessed (Miyake et al., 2000).

*Inhibition* was assessed by the number of commission errors in the Response Inhibition task [INHIB, S3 (go/nogo); Kaiser et al., 2010] and the “reading interference tendency” of the STROOP test.

*Shifting* was quantified by task switching speed in the Task Switching test (SWITCH; Gmehlin et al., 2012) as well as by the difference in working time for part B minus part A in the Trail-Making Test – Langensteinbach Version (TMT; Rodewald et al., 2012).

*Updating* was measured by the number of correct trials in the Digit Span Backwards task (DSB) from the Wechsler Adult Intelligence Scale – Fourth Edition (WAIS-IV; Petermann, 2012) and by the number of correct answers in the N-Back Verbal (NBV, S1 2-back, S3 3-back; Schelling and Schuri, 2009).

To assess the specificity of the impact of foreign language learning on EF, we included tasks from two domains that are less likely to be affected by foreign language learning. These domains cover linguistic functions (verbal fluency) and non-executive components (attention).

*Verbal fluency* was measured by the number of correct words in semantic (S2) and lexical (S4) fluency from the Vienna Verbal Fluency Test (WIWO; Jahn, 2016).

*Attention* was assessed by the logarithmic mean reaction time in the Alertness test, a subtest of the Perception and Attention Function Battery (WAFA; Sturm, 2006a), the number of correct trials in the Digit Span Forwards (DSF; Petermann, 2012), which can be considered as a measure of attention besides short-term memory span (Cambridge Cognition, 2023), and the working time in the TMT part A (TMT-A; Rodewald et al., 2012).

#### Language course outcomes

To evaluate the intervention, we assessed adherence to the language course and the homework and learning time at home. At the end of the course, participants anonymously filled out a course evaluation questionnaire. They reported their degree of motivation and satisfaction with the course and rated the quality of the lessons, the teacher, and the textbook on a Likert scale. The scale ranged from 0 to 4, with 4 indicating the highest agreement and 0 the strongest disagreement. The vocabulary test demanded to translate 108 words learned over the course into Spanish. If participants translated and wrote words correctly, they earned two points, making a total score of 216 possible. Only one point was awarded if the translated word had the correct meaning but was misspelled.

### Sample size

We pre-estimated the required sample size based on the first hypothesis and the primary outcomes (improvement of the LLG in either the STROOP naming interference tendency or in the WAFG between pre- and post-assessment compared to the WLCG) (Moher et al., 2010). Our calculation run on G*Power (version 3.1.9.2) resulted in 42 participants being required. We also considered a 30% drop-out rate (Pfenninger and Polz, 2018), making a total of 60 participants deemed necessary. We set the parameters in G*Power as follows: α = 0.05, corrected for multiple comparisons using the Bonferroni-Holm correction, power to detect significant differences of 0.8. Based on effect sizes found in some previous studies (Bak et al., 2016; Pfenninger and Polz, 2018), we considered a medium effect size of *d* = 0.25 to be reasonable (Cohen, 1988). At default, the correlation between repeated measures was set very conservatively at an *r* = 0.5. However, as mentioned above, we ended our study due to the Corona pandemic after 37 participants (ignoring drop-outs and non-adherence to the course) had completed the post-assessment. Since the correlation between repeated measures in the STROOP naming interference tendency and the WAFG was higher than estimated at *r* > 0.6 and higher correlations increase test power (Caldwell et al., 2022), G*Power allowed for a lower sample size of 34 participants, or 17 participants per group. Therefore, we decided to terminate our trial early in order to not to expose any participant to the risk of COVID-19 infection.

### Statistical analysis

The study team conducted all statistical analyses using IBM SPSS Statistics 26 (IBM Corporation: Armonk, NY, USA). We proceeded with the per-protocol dataset, including all participants who completed at least pre- and post-assessment and a minimum of 14 h of formal language instruction (> 62% of the total course duration; Bak et al., 2016). Data from two participants in the LLG were missing at 3 month follow-up due to the COVID-19 pandemic. We replaced these missing data with multiple imputation, assuming that they were at least missing at random. Primary and secondary outcomes were analyzed using 2 (group) x 3 (time) repeated measures analyses of variance (ANOVAs) with *group* as the between-subjects factor and *time-point of assessment* (pre-, post-, 3 month follow-up) as the within-subjects factor. In case of a significant interaction, we conducted *post hoc* tests to evaluate hypotheses 1 and 2. We predicted a significant *group x time interaction* and hypothesized that the LLG would outperform the WLCG at post- (hypothesis 1) or 3 month follow-up (hypothesis 2) relative to pre-assessment. All hypotheses were tested two-sided with adjustments made for multiple comparisons using the Bonferroni-Holm correction for primary outcomes. No adjustments for multiplicity were undertaken for secondary endpoints because, in smaller studies of an exploratory nature, rejection of the alternative hypothesis is more of a constraint than a type 1 error (Schoenfeld, 1980). Partial eta square (η_*p*_^2^) was applied as an effect size indicator.

To assess the robustness of the results, we investigated whether the short-term effects of foreign language learning on cognitive measures also applied to the WLCG after completing the control group program. To this end, the WLCG served as its control. We predicted a significant difference between the change scores from 3- to 4-month follow-up compared to the change scores from pre- to post-assessment of the WLCG using *t*-tests for dependent measures.

For the LLG, we performed exploratory subgroup analyses to evaluate whether effects of foreign language learning on cognitive outcomes would depend on different levels of CR (hypothesis 3), foreign language knowledge and usage (hypothesis 4), or baseline cognition (hypothesis 5). We also incorporated the vocabulary test score as a marker of learning success (Lim et al., 2020). Initially, we planned to conduct multiple regression analyses for hypothesis 3. However, we refrained from doing so since our sample was not large enough to meet the sample size requirement for multiple regression analysis. Instead, we created a correlation matrix between age, CR indices (CRI-Education, CRI-Working Activity, CRI-Leisure Time, and CRI-Index) (hypothesis 3), the LSBQ-score (hypothesis 4), the CFD-Index score (hypothesis 5; defined as independent variables in the further moderation analyses), and the vocabulary test score. We plotted these variables against change scores of the primary and secondary outcomes (defined as the dependent variable in the regression analysis). Change scores for each test were calculated by subtracting the post- from the pre-assessment result. Kendall’s tau-b was used as a correlation index, as this marker is generally recommended with small samples and a considerable number of tied ranks (Field, 2009). If the requirements for regression were met, we further analyzed whether group moderated the association between predictor and outcome to evaluate whether the significant association was specific to the LLG. Moderation models were run using model 1 of Hayes’s PROCESS macro v. 4.0 (2021).

## Results

### Participants

**FIGURE 1:**
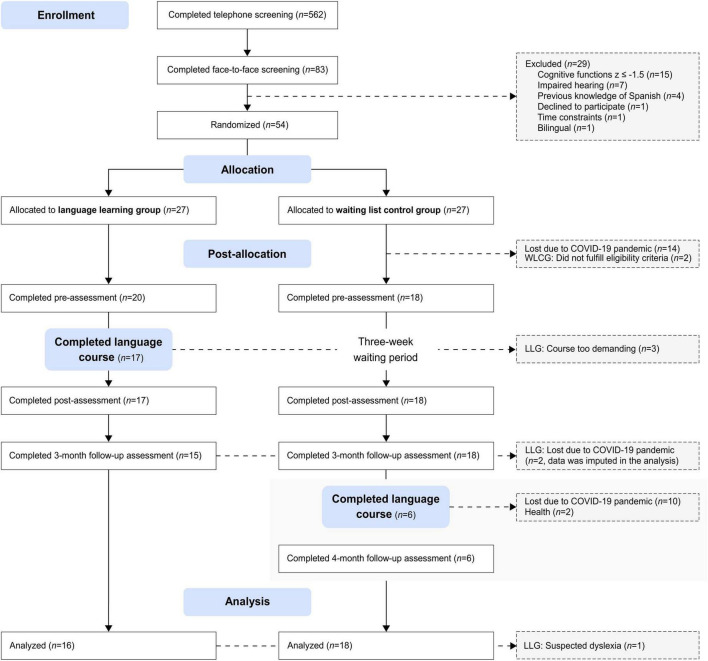
CONSORT flow diagram of participants.

A total of 54 participants were randomized into one of the two study arms. However, due to the COVID-19 pandemic and the consequent termination of the study, we lost 14 participants, who had already been assigned to one of the intervention groups but had not yet participated in the pre-assessment. Two participants in the WLCG were excluded from the trial because they did not meet eligibility criteria after the initial inclusion (CFD one subtest *z* ≤ −1.5, according to age and, where possible, sex, and education). Three participants from the LLG were excluded from the analysis because they did not finish the language course. One dropped out after 2 days, and two participants discontinued training after the first week because they experienced the course as too demanding. Another participant in the LLG was excluded from the final analysis due to suspected dyslexia. The final sample included in the analysis consisted of 34 participants who completed the intervention and the pre- and post-assessment. Figure 1 provides detailed information on participant flow and reasons for drop-out and losses due to the COVID-19 pandemic.

Demographic and language characteristics of participants are provided in Table 1. There were no notable differences between groups at baseline. The mean age was 69.47 (*SD* = 3.36) years. Nearly all participants were retired (*M* = 91.18%), the rest worked part-time at most. No participant scored below the cognitive threshold in any of the subtests of the CFD (*z* > −1.5). CR was in general high among both groups (*M* = 135.18, *SD* = 10.51). Regarding foreign language skills, participants can be considered monolingual on average (LSBQ: *M* = −3.57, *SD* = 1.73). None of them classified themselves as bilingual. Most reported having some basic knowledge of a Romanic language such as French and Italian (*n* = 26, 76.47%), and only a few had previous experience with Spanish (*n* = 11, 32.35%), e.g., having learned some basic vocabulary on holiday.

**TABLE 1:** Demographic and linguistic characteristics of participants in the two study arms.

Baseline characteristic	LLG (*n* = 16)		WLCG (*n* = 18)	
	Mean (*SD*)/*n* (%)	IQR (Q1, Q3)	Mean (*SD*)/*n* (%)	IQR (Q1, Q3)
Age *(years)*	69.00 (3.10)	(67.00, 70.00)	69.89 (3.61)	(66.00, 72.25)
Sex *(f)*	10 (62.50%)		10 (55.56%)	
Retired	15 (93.75%)		16 (88.89%)	
Handedness *(right)*	15 (93.75%)		16 (88.89%)	
CFD *(z-score)*^a^	0.61 (0.70)	(0.13, 0.99)	0.55 (0.59)	(0.04, 1.08)
CRIq-Index^b^	138.69 (10.83)	(131.75, 146.75)	132.06 (9.43)	(124.75, 137.25)
Education	125.50 (13.00)	(116.50, 138.50)	119.89 (12.56)	(107.75, 127.50)
Working activity	118.31 (11.72)	(107.25, 129.50)	114.28 (17.10)	(103.25, 128.00)
Leisure time	144.19 (15.18)	(130.25, 160.25)	138.39 (15.45)	(131.75, 149.25)
LSBQ^c^	−3.41 (1.60)	(−4.83, −2.58)	−3.72 (1.88)	(−5.00; −2.40)
Romanic languages	13 (81.25%)		13 (72.22%)	
Spanish	7 (43.75%)		4 (22.22%)	

CFD, cognitive functions dementia; CRIq, cognitive reserve index questionnaire; LLG, language learning group; LSBQ, language and social background questionnaire; WLCG, waiting list control group.

^a^*z*-score: ≤ −1.5 = below average; −1.5 < average > 1.5; ≥ 1.5 = above average.

^b^CRIq: ≤ 70 = low; 71−85 = low – medium; 86−114 = medium; 115−129 = medium – high; ≥ 130 = high.

^c^LSBQ: < −3.13 = monolingual; −3.13–1.23 = not strongly differentiated; > 1.23 = bilingual.

In the WLCG, *n* = 6 out of 18 participants completed the language course and subsequent assessment. Ten participants had to discontinue their course after the first week due to the COVID-19 pandemic, and two participants had to stop for health reasons. No study-related adverse events were reported.

### Primary outcomes

Data and results for tests of executive attention (primary outcomes), including exact *F*-, η_*p*_^2^- and *p* values for the two-way interactions between time and group of the 2 (group) x 3 (time) ANOVAs, are presented in Table 2. The analysis of the per-protocol and multiple imputation datasets revealed no significant interaction for the STROOP naming interference or the WAFG (*p* > 0.05). Additionally, there was no significant difference in the sub-analysis of the WLCG who completed the control group program (*p* > 0.05).

**TABLE 2:** Means (standard deviations) of primary outcome measures by trial arm and time point.

Outcome		LLG	WLCG	*F* (1, 32)	η_*p*_^2^	*p*	*p* ^1^
STROOP naming interference	Pre	211.44 (147.04)	253.61 (226.65)	0.016	<0.001	0.984	0.984
	Post	214.13 (115.53)	256.39 (214.21)				
	FU	220.50 (91.93)	255.89 (162.59)				
WAFG reaction time	Pre	517.22 (126.55)	524.35 (130.07)	0.974	0.030	0.383	0.766
	Post	522.13 (138.02)	497.95 (82.85)				
	FU	529.86 (156.84)	494.87 (95.42)				

*F*-values represent interaction effects between time and group calculated from 2 (group) x 3 (time) ANOVAs with repeated measures; FU, three-month follow-up assessment; LLG, language learning group; Pre, pre-assessment; Post, post-assessment; STROOP, stroop interference test – naming interference; WAFG, perception and attention function battery – divided attention; WLCG, waiting list control group.

^1^Bonferroni-Holm adjusted *p* value.

### Secondary outcomes

**FIGURE 2:**
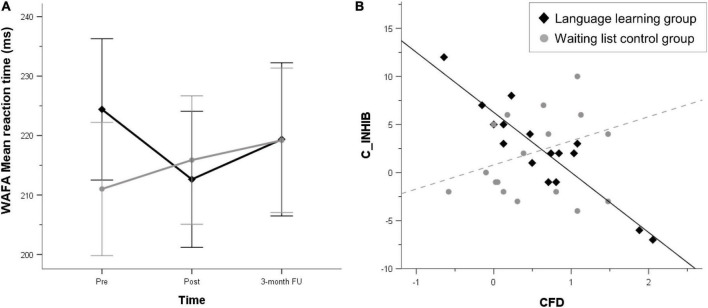
**(A)** Mean reaction time of the alertness subtest of the perception and attention function battery (WAFA) for each time point of assessment, divided by group. **(B)** Relationship between the change score between pre- and post-assessment of the Inhibition task (C_INHIB) commission errors and the index score of the cognitive functions dementia (CFD), divided by group.

Results of the secondary outcomes are presented in Table 3. We detected a significant interaction between time and group in the 2 (group) x 3 (time) ANOVA for the WAFA, *p* = 0.03, η_*p*_^2^ = 0.10. As discernible in Figure 2A, there was a slight group imbalance at pre-assessment, indicating worse performance for the LLG. To account for regression to the mean due to this imbalance, we calculated univariate Analyses of Covariance (ANCOVAs) for hypotheses 1 – short term effects – and 2 – long term effects of foreign language learning – separately, using the pre-assessment score as a covariate and the post- and the 3 month follow-up assessment score, respectively, as dependent variable (Vickers and Altman, 2001). Both for hypothesis 1, *F* (1, 31) = 2.55, *p* = 0.12, η_*p*_^2^ = 0.07, and for hypothesis 2, *F* (1, 31) = 3.64, *p* = 0.07, η_*p*_^2^ = 0.11, the ANCOVAs missed significance. These results indicate no significant difference between groups in intrinsic alertness after controlling for baseline imbalance, neither immediately nor 3 months after the intervention.

**TABLE 3:** Means (standard deviations) of secondary outcome measures by trial arm and time.

Outcome		LLG	WLCG	*F* (1, 32)	η_*p*_^2^	*p*
INHIB commission errors	Pre	14.19 (7.99)	13.22 (5.76)	1.599	0.048	0.214
	Post	11.75 (8.05)	11.06 (2.53)			
	FU	10.19 (6.51)	11.50 (4.48)			
STROOP reading interference	Pre	104.88 (99.25)	142.72 (99.78)	0.030	0.001	0.970
	Post	109.00 (70.90)	150.44 (100.61)			
	FU	104.88 (69.42)	149.67 (106.07)			
SWITCH switching speed	Pre	199.44 (208.26)	212.72 (195.95)	0.710	0.022	0.495
	Post	150.31 (180.78)	206.06 (182.37)			
	FU	160.56 (143.77)	198.33 (210.89)			
TMT working time part B-A	Pre	21.09 (8.74)	26.71 (22.21)	0.418	0.013	0.660
	Post	15.50 (7.86)	18.60 (9.57)			
	FU	20.46 (15.20)	21.19 (17.16)			
DSB correct trials	Pre	8.69 (1.62)	9.33 (1.94)	0.476	0.015	0.624
	Post	8.69 (1.70)	9.50 (2.04)			
	FU	9.63 (2.09)	9.94 (2.18)			
NBV S1 number correct	Pre	12.69 (2.06)	11.39 (2.50)	1.566	0.047	0.217
	Post	12.63 (1.78)	11.50 (2.68)			
	FU	12.44 (2.19)	12.28 (2.35)			
NBV S3 number correct	Pre	10.06 (2.17)	8.67 (2.61)	0.567	0.017	0.570
	Post	10.00 (3.01)	9.17 (2.87)			
	FU	10.50 (1.55)	8.83 (3.20)			
WIWO S2 number correct	Pre	43.63 (6.80)	38.56 (7.72)	0.460	0.014	0.583
	Post	40.94 (7.63)	38.44 (7.96)			
	FU	43.94 (9.45)	40.56 (8.89)			
WIWO S4 number correct	Pre	23.38 (7.59)	19.50 (6.96)	0.365	0.011	0.696
	Post	25.69 (8.75)	20.06 (7.48)			
	FU	25.00 (8.19)	20.17 (6.08)			
WAFA reaction time	Pre	224.40 (23.61)	211.01 (23.10)	3.692	0.103	**0**.030*
	Post	212.63 (17.16)	215.88 (26.30)			
	FU	219.36 (25.41)	219.20 (25.22)			
DSF number correct trials	Pre	10.06 (1.18)	9.39 (1.85)	0.016	0.001	0.984
	Post	9.94 (1.12)	9.28 (1.99)			
	FU	10.25 (1.61)	9.67 (1.75)			
TMT working time part A	Pre	14.91 (2.59)	15.79 (3.08)	0.351	0.011	0.705
	Post	14.71 (2.05	16.24 (5.45)			
	FU	14.45 (3.43)	16.18 (3.70)			

*F*-values represent interaction effects between time and group calculated from 2 (group) x 3 (time) ANOVAs with repeated measures; DSB, digit span backwards; DSF, digit span forwards; FU, three-month follow-up assessment; INHIB, response inhibition task; NBV, N-back verbal; LLG, language learning group; Post, post-assessment; Pre, pre-assessment; STROOP, stroop interference test – reading interference; SWITCH, task switching test; TMT, trail-making test – Langensteinbach version; WAFA, perception and attention function battery – alertness; WIWO, Vienna verbal fluency test; WLCG, waiting list control group.

* p < 0.05. Significant interaction effects are highlighted in bold.

In the sub-analysis of the WLCG, who completed the control group program, there likewise were no significant differences between the predefined change scores (*p* > 0.05). Yet, there was also a slight, but non-significant, *t* (5) = −2.38, *p* = 0.06, improvement in the WAFA from 3- to 4-month follow-up compared to the change score from pre- to post-assessment, suggesting that the WLCG also tended to benefit from foreign language learning in intrinsic alertness.

Note that in our protocol, we intended to conduct an intention-to-treat analysis in addition to the per-protocol analysis. However, the intention-to-treat analysis was meant to test the robustness of the results (Tripepi et al., 2020). We refrained from doing so as there were no significant results for either the primary or secondary endpoints.

### Moderation analysis

In a first step, we ran Kendall’s tau-b correlations among participants in the LLG to identify significant associations between predictors (age, indices of the CRIq, LSBQ score, CFD index score, vocabulary test) and outcomes (change scores between pre- and post-assessment in primary and secondary outcomes). The correlation matrix is depicted in Table 4. Significant associations are highlighted in bold. For significant associations, we further conducted moderation analyses with group as moderator to determine whether the associations in the LLG differed significantly from those in the WLCG.

**TABLE 4:** Correlation matrix of measures of the language learning group (*n* = 16).

Variable	1	2	3	4	5	6	7	8
1. Age	—							
2. CFD	0.30	—						
3. CRIq-Index	0.09	0.05	—					
4. CRIq-Education	0.18	0.03	**0**.57**	—				
5. CRIq-Working activity	-0.07	0.03	0.36	0.29	—			
6. CRIq-Leisure time	0.17	0.11	0.30	0.03	-0.30	—		
7. LSBQ	0.12	0.29	0.31	0.23	0.25	0.12	—	
8. Vocabulary test	-0.09	0.14	0.25	0.00	0.06	0.31	0.38*	—
C_STROOP_N	0.08	0.24	0.00	0.03	0.10	-0.11	0.06	0.13
C_WAFG	0.23	-0.33	0.03	0.14	0.18	-**0**.44*	-0.20	-**0**.44**
C_INHIB	-0.20	-**0**.64***	-0.09	-0.04	0.13	-0.23	-0.19	-**0**.38*
C_STROOP_R	0.10	0.13	-0.29	-0.06	-0.21	-0.07	-0.02	0.01
C_SWITCH	0.08	0.18	-0.16	-**0**.39*	-0.04	-0.05	0.10	0.07
C_TMT B-A	0.05	0.01	0.03	0.19	-0.06	0.03	0.20	0.10
C_DSB	-0.20	-0.08	0.01	0.16	0.29	-0.26	0.19	-0.18
C_NBV S1	0.14	-0.02	-0.04	-0.18	0.10	0.02	-0.09	-0.22
C_NBV S3	-0.11	-0.14	-0.13	-0.18	0.00	0.08	-0.13	0.29
C_WIWO S2	-0.05	0.08	-0.07	0.01	-0.10	0.01	-0.14	-0.01
C_WIWO S4	0.23	0.28	0.02	0.10	0.26	-0.16	0.34	-0.13
C_WAFA	-0.03	-0.01	0.03	-0.01	-0.18	0.17	0.22	0.06
C_DSF	0.13	0.08	-0.06	0.27	0.14	-0.21	-0.09	-0.09
C_TMT-A	-0.33	-0.16	-0.16	-0.23	-0.11	-0.20	-**0**.50**	-0.20

Kendall’s tau-b correlations between baseline variables and the vocabulary test score, and change scores of primary and secondary outcomes between pre- and post-assessment; CFD, cognitive function dementia; CRIq, measures of the cognitive reserve index questionnaire; LSBQ, language and social background questionnaire; C_DSB, change score of the digit span backwards; C_DSF, change score of the digit span forwards; C_INHIB, change score of the response inhibition task; C_NBV S1, change score of the NBV N-back verbal S1; C_NBV S3, change score of the NBV N-back verbal S3; C_STROOP_N, change score of the stroop interference test – naming interference; C_STROOP_R, change score of the STROOP – reading interference; C_SWITCH, change score of the task switching test; C_TMT B-A, change score of the trail-making test – Langensteinbach version part B – part A; C_TMT-A, change score of the trail-making test – Langensteinbach version part A; C_WAFA, change score of the perception and attention function battery – alertness; C_WAFG, change score of the perception and attention function battery – divided attention; C_WIWO S2, change score of the Vienna verbal fluency test S2; C_WIWO S4, change score of the Vienna verbal fluency test S4. * *p* < 0.05, ** *p* < 0.01, *** *p* < 0.001. All significant correlations are highlighted in bold.

The moderation model of the prediction of the change score of the WAFG by the CRIq Leisure time sub-score, moderated by group missed significance, *F* (3, 30) = 2.40, *p* = 0.09, *R*^2^ = 0.23. Also, the model of the change score of the TMT-A predicted by the LSBQ, *F* (3, 30) = 3.17, *p* = 0.18, *R*^2^ = 0.10, and of the SWITCH predicted by the CRIq Education sub-score, *F* (3, 30) = 1.96, *p* = 0.14, *R*^2^ = 25, were not significant. However, for the change score of the INHIB predicted by the CFD, the overall model was significant: *F* (3, 30) = 36.46, *p* < 0.001, *R*^2^ = 0.43. Figure 2B shows the relationship between predictor and outcome divided by group. Group and the CFD score did not significantly predict the change score of the INHIB (*p* > 0.05).

However, the interaction term was highly significant *b* = 8.75, *t* (30) = 3.86, *p* < 0.001. The regression slope was only significant for the LLG, *b* = −6.24, *t* (30) = −10.39, *p* < 0.001; WLCG: *b* = 2.51, *t* (30) = 1.15, *p* = 0.26, meaning that for the WLCG, the CFD did not predict change from pre- to post-assessment in the INHIB. In the LLG, lower CFD scores predicted stronger improvement from pre to post in the INIHB.

The vocabulary test score was not entered in the moderation analyses as only participants from the LLG conducted a vocabulary test. For the vocabulary test score, there was a significant negative correlation with the LSBQ, τ_b_ = −0.38, *p* = 0.01, indicating better performance in the vocabulary test among individuals with higher LSBQ scores. The vocabulary test score also correlated strongly negatively with the change score of the WAFG, τ_b_ = −0.44, *p* = 0.005, implying that individuals with lower vocabulary scores showed more improvement in the WAFG. And there was a significant negative correlation with the change score of the INHIB, τ_b_ = −0.38, *p* = 0.02, again indicating a more marked improvement in the INHIB for individuals with lower vocabulary test scores.

### Language course outcomes

#### Adherence

Overall, adherence to the intervention was high, with a mean of 14.63 (*SD* = 0.50) course days attended. Most participants (62.5%) completed the full course duration.

#### Homework and learning time

Participants spent *M* = 12.25 (*SD* = 5.79) hours with additional homework and learning activities at home. In this regard, we detected considerable disparities between participants (range = 4.42–23.58 h). *Post hoc* analyses indicated no significant correlation between time spent with additional homework and learning activities at home and performance on cognitive variables at baseline (*p* > 0.05).

#### Course evaluation

Participants’ opinions on the language course were based on the total proportion of the sample that completed the language course and submitted an evaluation. Since the evaluation was anonymous, two participants who dropped out from the language course but submitted an evaluation are included here. Overall, the responses were very positive, with the teacher and the quality of the lessons being rated highest [lesson: *M* = 3.88 (*SD* = 0.23), teacher: *M* = 3.98 (*SD* = 0.08)]. Also, opinions about the textbook, participants’ motivation, and satisfaction were favorable [textbook: *M* = 3.65 (*SD* = 0.59), motivation: *M* = 3.32 (*S*D = 0.59), satisfaction *M* = 3.64, (*SD* = 0.45)].

Additionally, in open format questions, participants indicated that they were most positive about the quality of teaching. For example, one participant said, “[The teacher] gave clear and understandable explanations and always patiently corrected mistakes. I would very much like to continue the course with her.” Aspects participants disliked or would recommend for improvement were mainly related to the textbook, the classroom, the intensity and duration of the course, and the different learning paces within the groups. One participant perceived the textbook as somewhat complicated, e.g., when searching for vocabulary. Some participants would also have preferred to learn more about Spain. The classroom was perceived as too small to create a good learning atmosphere and allow group work. Therefore, the second half of the participants were taught in a larger room. Regarding the intensity of the course, one participant claimed that the course was too intensive. Two participants felt the course was too short, and another would have liked to continue, but only twice a week. Four participants rated the differences in learning pace between participants and prior knowledge of related languages (e.g., French) as too large. Some considered the pace too fast and would have welcomed more time for repetition, whereas another participant said the lessons could have proceeded more quickly.

#### Vocabulary test

The mean score of the post-intervention vocabulary test was *M* = 160.88 (*SD* = 41.17), indicating that, on average, participants had acquired a basic Spanish vocabulary. However, again, we detected major differences between participants, with scores ranging from 84 to 210. *Post hoc* analysis revealed no significant correlation between the result of the vocabulary test and the homework and learning time (τ_b_ = −0.04, *p* = 0.82).

## Discussion

In older adults aged 65–80 years, a three-week-long intensive Spanish course for beginners did not elicit improvements in executive attention relative to a passive control group neither immediately nor 3 months after the training. We also saw no significant increase in our secondary outcomes encompassing measures of EF, verbal fluency, and attention. Intrinsic alertness improved in the LLG compared to the WLCG after participation in the course. However, when we considered differences between groups in the pre-measurement, this effect failed to reach significance. The sub-analysis of the WLCG who completed the control group program also did not reveal any significant change in cognitive performance after completing the course. However, examining factors that influenced cognitive responses in language learners, moderation analyses showed that differences in global cognition at baseline predicted changes in response inhibition. The lower the language learners’ baseline cognition, the greater were the gains in response inhibition. This association was not evident in individuals of the WLCG. Similarly, correlation analyses revealed that lower vocabulary test scores as a marker of learning were associated with more remarkable improvement in response inhibition after the end of the course. This relationship could be due to higher cognitive engagement among those with lower vocabulary test scores.

### Primary and secondary outcomes

For our primary outcome measures – the Stroop naming interference and the WAFG – representing two sub-domains of executive attention, namely selective and divided attention, respectively, we did not find beneficial effects of foreign language learning. Also, we did not observe any general training-related gains in our secondary outcome measures immediately or 3 months after the course. As participation in the course was generally high, and learners’ assessments of the teacher and the quality of the lessons were generally very positive, we contend that the overall non-significant results in our primary and secondary outcomes are not the result of a lack of participant engagement. Participants also stated that they were generally motivated to participate in the training. In addition, the outcome of the vocabulary exam demonstrates that participants gained fundamental knowledge while taking the course.

The lack of improvement is consistent with findings from previous studies on the cognitive benefits of foreign language learning (Ramos et al., 2017; Ware et al., 2017; Valis et al., 2019; Berggren et al., 2020; Kliesch et al., 2021). Our results may underline the notion of missing far-transfer effects of foreign language acquisition on supposedly affected cognitive domains. The difficulty of detecting far-transfer effects is a considerable problem accompanying many studies trying to demonstrate the cognitive benefits of a particular intervention. In general, most cognitive gains rarely extend beyond the specific domain being practiced. An example of studies showing near-transfer effects are cognitive training studies in which a particular cognitive domain is intentionally trained. Foreign language learning studies are referred to as far transfer because they do not specifically target any particular cognitive domain. In a second-order meta-analysis, Sala et al. (2019) found that far transfer effects of cognitive training were either small or non-existent. Thus, it might be that far-transfer effects of foreign language training are minor, if existent, and therefore difficult to detect.

However, some studies oppose the assumption of missing far transfer effects of foreign language learning (Bak et al., 2016; Pfenninger and Polz, 2018; Bubbico et al., 2019; Wong et al., 2019; Long et al., 2020; Meltzer et al., 2021). For example, a fairly recent RCT conducted by Meltzer et al. (2021) found significant gains in the naming interference condition of the Stroop task and working memory following 16 weeks of Spanish instruction. The reason we did not discover benefits in the executive attention domain equally in either selective or divided attention could be attributed to the format of our language course. Unlike Meltzer et al. (2021), we did not apply app-based language training. This kind of training allows to better meet participants at their performance level than a group-based face-to-face program and thus might have led to higher cognitive engagement in Meltzer et al.’s study.

Among language learners, we also, for instance, observed an improvement in intrinsic alertness after the course, which did not hold significance after controlling for differences between groups at pre-test. Meltzer et al. (2021) similarly found a medium, albeit non-significant, effect on processing speed in foreign language learners compared to a passive control group, which, like intrinsic alertness, measures intensity-related aspects of attention. Intrinsically maintained tonic alertness is a core function of the cingulo-opercular network, which is as part of the executive attention network also responsible for monitoring behavior, task-set maintenance, and salience detection (Sadaghiani and D’Esposito, 2015). The cingulo-opercular network is among the main cognitive networks that degenerate with aging. In addition, recent behavioral and functional data suggest that the bilingual advantage in older adults may be particularly pronounced in the alerting dimension (Dash et al., 2019). Future research should thus test this hypothesis even though our data did not show a substantial impact of learning a foreign language on intrinsic alertness.

### Moderation analyses

The moderation analyses showed no significant impact of the CR indices or the foreign language proficiency and usage index on change scores of cognitive outcome measures. The prediction of the change score in divided attention by the CRIq Leisure time sub-score in the moderation model fell short of significance. The significant correlations between the change score in task switching and the CRIq education sub-score as well as between the change score in information processing speed and self-rated foreign language proficiency and usage also remained non-significant when entered into the moderation model. These insignificant findings are surprising given that in Mondini et al. (2016), individuals with mild to moderate probable dementia and lower CR improved more in global cognition after cognitive training than those with higher CR. Similarly, Long et al. (2020) demonstrated that healthy younger and older language learners who had lower Gaelic knowledge and consequently were placed in a Gaelic beginner class improved more on attentional switching after intensive language training than those in advanced courses.

However, our moderation analysis using baseline cognitive performance as a predictor was significant. Those Spanish learners who scored lower on baseline cognition were more likely to improve in response inhibition measured by a go/nogo task paradigm than language learners with higher baseline cognitive performance.

The baseline performance-dependent improvement in response inhibition is striking in several ways. First, it is in line with a recent publication by Kliesch et al. (2021). They detected that in healthy older adults, lower baseline cognition was related to more substantial gains in cognitive outcomes exclusively in language learners and most intensely for WM accuracy. In contrast, the authors found no such significant associations in the active and passive control groups. Their results underline the unique benefits of foreign language learning for subjects with lower baseline cognition who are more vulnerable to cognitive decline. According to the CR hypothesis, these individuals might benefit more from engaging in cognitively stimulating activities as their CR is lower and, therefore, their brains have more room for improvement (Valenzuela and Sachdev, 2006). The reason we did not find a significant moderation model in the proxy measures of CR (education, occupation, and leisure activity) but only for global cognition as a predictor may have the reason that global cognition is usually considered a more direct representation of CR (Opdebeeck et al., 2016). As the CR hypothesis predicts, individuals with a lower level of cognitive functioning are more likely to develop dementia than those with better cognitive performance (Whalley et al., 2004). Thus, the influence of CR on cognitive outcomes might have just been more readily apparent with a more direct representation of CR.

Second, while no study in this field on healthy older adults has included a go/nogo task paradigm, response inhibition may be promising as a core cognitive domain affected by foreign language learning. The go/nogo task paradigm is the only one, apart from the Stop-Signal Task, to show a marked age-related deficit in inhibition compared to other tasks frequently applied in bilingual research, such as the Stroop, Flanker, or Simon task (Rey-Mermet and Gade, 2018). According to early work by Persad et al. (2002), inhibition deficits are a precocious hallmark of cognitive decline. Also, inhibition deficits may underlie impairments in other cognitive domains, such as attention and episodic memory. Consequently, older adults are less able to suppress dominant responses while simultaneously maintaining two task sets (go- vs. nogo-stimulus). Applied to the context of foreign language learning, learners’ brains may be trained to suppress dominant responses (i.e., their mother tongue) while processing the less dominant stimulus (i.e., the foreign language). This view also aligns with the Adaptive Control Hypothesis. According to this hypothesis, language learners in a dual-language context, which is commonly given in a classroom setting, require selective response inhibition when communicating in the less dominant language (Green and Abutalebi, 2013), since both languages are always active in the brain (Kroll et al., 2015). Learning a foreign language therefore, may be among the most effective interventions to prevent cognitive decline and dementia, as it is likely to directly affect response inhibition, which is probably fundamental to healthy cognitive aging (Persad et al., 2002).

We interpret our finding in the context of higher cognitive engagement among those with lower global cognitive baseline scores. On the one hand, we derived this assumption from a highly significant negative association between the vocabulary test result and the change score in response inhibition. According to this correlation, individuals with lower vocabulary test scores improved more in response inhibition. The fact that there was no significant correlation between additional hours spent with homework activities and the vocabulary test result rules out the plausible explanation that individuals with lower test results just engaged less. It may instead indicate greater cognitive strain among those same individuals. On the other hand, we derived our conclusion from qualitative feedback from the attendees. Their feedback supports the view that participants were differently challenged: Four participants judged the differences in their learning rates and their past exposure to related languages (for example, French) as excessively great. While one participant felt the sessions could have gone more rapidly, others thought the pace was too fast and would have appreciated additional time for repetition. Aside from large differences in global cognition between participants, disparities in previous foreign language skills, e.g., in the Romanic languages, may imply that participants were differentially challenged. Even though we made sure that previous skills in Romanic languages were low (< B1 as defined by questions derived from the Joint European Reference Frame for Languages) and that participants were not bilingual, there was a significant correlation between the vocabulary test score and the LSBQ score. Individuals who had fewer foreign language skills, as assessed by the LSBQ, also performed worse on the vocabulary test. Therefore, these individuals had less prior knowledge to draw on during the course and thus may have been more challenged.

### Strengths and limitations

Several strengths and limitations of our study design and analysis must be respected when interpreting our results.

We conducted the first study to implement a short but intensive foreign language course in a randomized-controlled design. Moreover, we essentially implemented the recommendations that Ware et al. (2021) established regarding the conduct of foreign language studies. First, we chose our primary outcomes theory- and evidence-based. Second, we included a broad test battery of change-sensitive cognitive outcomes from the domains of executive attention, EF, verbal fluency, and attention. Third, we conducted an objective foreign language assessment of the language taught to exclude bilingual individuals and those with more than basic knowledge of Spanish, who are thus not suitable for an A1.1 course for beginners. Furthermore, we excluded participants with suspected cognitive impairment using a comprehensive neuropsychological test battery, and we administered a vocabulary test at the end of the intervention to assess learning gains.

One drawback is our modest sample size. Due to the COVID-19 pandemic, the last language course, for which ten participants were scheduled, could not be conducted. Thus, together with the missing participants from the WLCG, the pandemic outbreak resulted in a total loss of 20 participants. Hundreds of clinical trials were affected by the pandemic^2^. The fact that these studies could not all reach their initially estimated sample size should be considered when evaluating them. However, we claim that our sample size was still large enough to detect medium effects in one of our primary outcomes, as the correlation between repeated measures was higher than pre-estimated in our study protocol. Therefore, even with a smaller sample size, it was still feasible to detect medium effects in our primary outcomes, an effect size also found in other related studies in the field (Bak, 2016; Pfenninger and Polz, 2018; Wong et al., 2019). Furthermore, no small effect was apparent for most of the primary and secondary outcomes, making it unlikely that 20 more participants would have materially altered the results. It also should be noted that the current study was meant to be exploratory to identify cognitive domains and tasks altered by foreign language learning. The cognitive effects of the early stages of foreign language acquisition in older adults have received little attention thus far. Therefore, preliminary studies must first be carried out before conducting large-scale studies.

One could also argue that our intervention was not long or the proficiency level attained was not high enough to elicit more noticeable cognitive changes. However, we posit that Bak et al. (2016) and Long et al. (2020) found an improvement in attentional switching after even 1 week of high-frequency foreign language training. Moreover, lengthier interventions, e.g., 8 months (Ramos et al., 2017; Kliesch et al., 2021), with thus higher proficiency level attained, were not necessarily associated with cognitive improvements. Additionally, none of the low-frequency courses resulted in significant cognitive gains (Ware et al., 2017; Bubbico et al., 2019; Valis et al., 2019). Consequently, it seems reasonable that the frequency of training held over a specified period is more relevant than its actual length or the proficiency level reached. In addition, from cognitive training studies, it is well known that even significantly less training than delivered in our study can lead to significant improvements in cognition in older adults [Borella et al., 2010: three training sessions; Carretti et al., 2013: six training sessions completed within 2 weeks; Nouchi et al., 2012: game playing for 15 min per day, at least 5 days per week for 4 weeks; see the review of Kelly et al. (2014) for more examples of studies]. Furthermore, a very recent publication showed that benefits of cognitive training reach a plateau after 12−14 h of training (Belleville et al., 2021), a number of training sessions we provided in our study. In sum – even though our study differs from other related studies in terms of intervention, design, outcomes, or population, there is sound evidence in the literature that the dosage of our intervention in terms of frequency and proficiency level achieved was high enough to affect cognition generally. Additionally, we actually found an improvement in response inhibition in individuals with lower baseline cognition. Another recent study also supports the importance of baseline cognition for cognitive improvements related to foreign language learning (Kliesch et al., 2021). Given that our sample was generally well educated, as can be inferred from the education sub-score of the CRIq, and higher education is linked to better cognitive performance (Opdebeeck et al., 2016), cognitive improvements could have been obscured among those with higher education. Concluding from our results, we thus consider finding a way to adequately address all participants intellectually in a treatment plan more crucial than its actual dosage.

Another caveat is that we did not include an active comparator. We justify this decision on economic grounds. As previous studies have not conclusively identified cognitive domains addressed by foreign language learning, it is essential to evaluate these before adjusting for alternative explanations (Puhan et al., 2008). Thus, we cannot rule out that the benefit in response inhibition after foreign language learning for subjects with lower baseline cognition is due to general cognitive stimulation through social interaction (Kelly et al., 2017). Excluding alternative explanations was also not the aim of our exploratory study, as we first wanted to uncover cognitive domains and tasks potentially impacted by foreign language learning. However, one indication that the effect found could be due to foreign language learning is that the vocabulary test score, which is highly language-related, strongly correlated with changes in response inhibition. Still, we urge that future studies should include an active control group to account for other possible explanations.

## Conclusion

We did not observe any overarching benefits of foreign language learning in executive attention or EF. However, we found evidence that foreign language learning may improve response inhibition, a domain particularly affected by cognitive aging, in older adults with lower global cognitive baseline levels. Based on our findings, future studies should consider individual differences and target participants with lower baseline cognition, who are thus more vulnerable to cognitive decline and dementia. Systematically selecting participants with lower baseline performance and less foreign language experience might be similar to physical training studies that exclude subjects who are too physically fit for a particular intervention (Jansen et al., 2021). In parallel, interventions must be more flexible to learners’ abilities and needs. For example, applying technology-assisted learning of grammatical rules and vocabulary, e.g., using an app (Meltzer et al., 2021), and individually guided and planned by a specialized teacher, would better allow meeting participants at their performance level. At the same time, frustration due to excessive demands, which is more likely in a group setting with different learning paces, would be avoided. If combined with informal group activities covering recreational topics such as culture or traveling, social aspects of language learning would also be addressed to promote individual well-being and motivation through social integration (Pfenninger and Singleton, 2019). In view of these aspects, we might find more conclusive evidence of the impact of foreign language learning on executive attention and EF. This presupposes that we are able to determine the precise training dose, both in terms of length, frequency, and difficulty, and cognitive domains addressed by foreign language learning. Therefore, we will need well-controlled studies with large sample sizes in this field to delineate the specificity of the effects of foreign language learning on cognition as opposed to other cognitively stimulating interventions. Meanwhile, as much as other cognitively enriching leisure time activities that are unlikely to cause harm foreign language learning should be recommended to strengthen CR.

## Data availability statement

The raw data supporting the conclusions of this article will be made available by the authors, without undue reservation.

## Ethics statement

This study involving human participants was reviewed and approved by the Ethics Committee of the Faculty of Behavioral and Empirical Cultural Studies of the Ruprecht Karl University of Heidelberg, reference number, 2018/1-2, on 10 January 2019. The participants provided their written informed consent to participate in this study.

## Author contributions

JG, PM, and BT conceived the study. SA contributed to the design of the study. JG conducted the study and wrote the manuscript. SA, PM, and BT critically reviewed the manuscript for relevant intellectual content. All authors read and approved the final manuscript.

## Footnotes

1
https://www.sealedenvelope.com/

2
https://www.transparimed.org/single-post/2020/05/08/Discontinued-clinical-trials-COVID
